# Atomic Layer Deposition of Pt Nanoparticles within the Cages of MIL-101: A Mild and Recyclable Hydrogenation Catalyst

**DOI:** 10.3390/nano6030045

**Published:** 2016-03-09

**Authors:** Karen Leus, Jolien Dendooven, Norini Tahir, Ranjith K. Ramachandran, Maria Meledina, Stuart Turner, Gustaaf Van Tendeloo, Jan L. Goeman, Johan Van der Eycken, Christophe Detavernier, Pascal Van Der Voort

**Affiliations:** 1 Department of Inorganic and Physical Chemistry, Center for Ordered Materials, Organometallics and Catalysis (COMOC), Ghent University, Krijgslaan 281-S3, B-9000 Ghent, Belgium; norinibinti.tahir@ugent.be; 2 Department of Solid State Sciences, Conformal Coatings on Nanomaterials (CoCooN), Ghent University, Krijgslaan 281-S1, B-9000 Ghent, Belgium; jolien.dendooven@ugent.be (J.D.); ranjith.karuparambilramachandran@ugent.be (R.K.R.); christophe.detavernier@ugent.be (C.D.); 3 EMAT, University of Antwerp, Groenenborgerlaan 171, B-2020 Antwerp, Belgium; maria.meledina@uantwerpen.be (M.M.); stuart.turner@uantwerpen.be (S.T.); staf.vantendeloo@uantwerpen.be (G.V.T.); 4 Department of Organic and Macromolecular Chemistry, Laboratory for Organic and Bioorganic Synthesis, Ghent University, Krijgslaan 281-S4, B-9000 Ghent, Belgium; jan.goeman@ugent.be (J.L.G.); johan.vandereycken@ugent.be (J.V.E.)

**Keywords:** metal organic frameworks, atomic layer deposition, platinum, hydrogenation

## Abstract

We present the *in situ* synthesis of Pt nanoparticles within MIL-101-Cr (MIL = Materials Institute Lavoisier) by means of atomic layer deposition (ALD). The obtained Pt@MIL-101 materials were characterized by means of N_2_ adsorption and X-ray powder diffraction (XRPD) measurements, showing that the structure of the metal organic framework was well preserved during the ALD deposition. X-ray fluorescence (XRF) and transmission electron microscopy (TEM) analysis confirmed the deposition of highly dispersed Pt nanoparticles with sizes determined by the MIL-101-Cr pore sizes and with an increased Pt loading for an increasing number of ALD cycles. The Pt@MIL-101 material was examined as catalyst in the hydrogenation of different linear and cyclic olefins at room temperature, showing full conversion for each substrate. Moreover, even under solvent free conditions, full conversion of the substrate was observed. A high concentration test has been performed showing that the Pt@MIL-101 is stable for a long reaction time without loss of activity, crystallinity and with very low Pt leaching.

## 1. Introduction

Metal Organic Frameworks (MOFs) are a class of porous crystalline materials consisting of discrete inorganic and organic secondary building units. Due to their exceptionally high porosity, pore volume, large surface area and chemical tunability and flexibility, they have already been examined in a wide range of areas such as gas storage and separations, sensing, drug delivery, ion exchange and as heterogeneous catalysts [1,2,3]. When used as a heterogeneous catalyst, MOFs can be examined as such or can be utilized as a support to stabilize catalytic active sites [4]. Besides the encapsulation of homogeneous complexes [5] and polyoxometalates [6], there is a growing research interest towards the embedding of nanoparticles (NPs) into MOFs [7]. The size, shape and orientation of the NPs can be controlled by adjusting the pore size and shape of the MOFs. Moreover, the nanopores of the MOFs can be used as templates for the synthesis of monodispersed NPs. Thus far, mainly Pd [8], Au [9], Ru [10], Cu [11], Pt [12], Ni [13] and Ag [14] NPs have been incorporated into MOFs through incipient wetness impregnation, colloidal deposition, solid grinding and chemical vapor deposition.

In recent years, atomic layer deposition (ALD) has gained renewed attention as a flexible method for tailoring mesoporous materials toward specific catalytic applications [15,16,17]. ALD is a self-limited deposition method that is characterized by alternating exposure of the substrate to vapor-phase precursors to grow oxides, nitrides, sulfides and (noble) metals [18]. The self-limiting nature of the chemical reactions yield atomic level thickness control and excellent uniformity on complex three-dimensional supports such as mesoporous materials. In noble metal ALD, islands are often formed at the start of the ALD process instead of continuous layers. This island growth can be used advantageously to synthesize noble metal NPs on large surface area supports. Several authors have demonstrated the successful synthesis of highly dispersed Pt NPs with narrow size distributions [19,20,21,22,23,24]. Despite the unique advantages of MOFs as a scaffold in catalytic systems, noble metal ALD in MOFs has not yet been explored. The main challenge for ALD in MOFs is the slow diffusion of the chemical precursors within Ǻngstrom sized pores [25,26,27]. Therefore, Hupp and coworkers fabricated a Zr-based NU-1000 MOF with large 1D hexagonal channels (~30 Ǻ) and successfully realized the ALD-based incorporation of acidic Al^3+^ and Zn^2+^ sites [28] and catalytically active cobalt sulfide [29]. Computational efforts provided mechanistic insight in the interaction of the ALD precursors with the MOF nodes [30]. Very recently, Jeong *et al.* reported the ALD of NiO within the framework of MIL-101-Cr (MIL = Materials Institute Lavoisier) [31]. This MIL-101 framework consists of two types of pores with inner pore diameters in the low mesoporous regime (~25–35 Ǻ) and is thermally stable up to 300 °C. The MIL-101 framework is built up by Cr_3_O-carboxylate trimers and terephthalate linkers with octahedrally coordinated metal ions binding terminal water molecules (see Figure S1) [32]. Hwang *et al.* [33] have demonstrated that these coordinated water molecules can be easily removed by a thermal treatment under vacuum at a temperature of 150 °C, creating coordinatively unsaturated sites (CUSs) which could be used, besides the Cr_3_O trimers, as initial binding sites for the anchoring of nanoparticles. Because of these advantages, MIL-101-Cr was selected in this work as the MOF host for catalytically active Pt NPs synthesized by ALD. It is shown that Pt ALD results in highly dispersed, uniformly sized NPs embedded within both the small and larger MIL-101 pores. In addition, this paper reports on the catalytic properties of the Pt@MIL-101 material in the hydrogenation of different linear and cyclic olefins at room temperature.

## 2. Results and Discussion

### 2.1. Characterization of Pt@MIL-101-Cr

#### 2.1.1. X-Ray Diffraction, Nitrogen Adsorption and Determination of the Pt Loading

The Pt loading of each Pt@MIL-101-Cr material was determined by means of XRF (see Table 1). As expected, an increasing number of ALD cycles resulted in a higher Pt loading. For the 40, 80 and 120 ALD cycles, a Pt loading of respectively 0.21, 0.30 and 0.35 mmol·g^−1^ was obtained. The latter sample was used for the catalytic evaluation.

**Figure 1:**
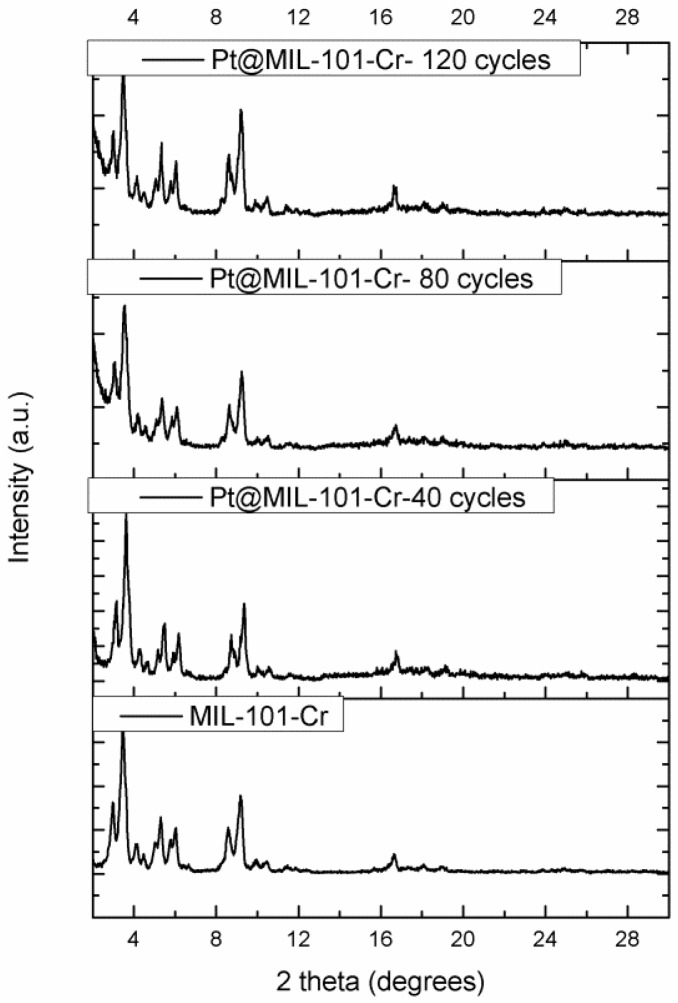
X-ray powder diffraction (XRPD) patterns of MIL-101-Cr and the obtained Pt@MIL-101-Cr materials (MIL = Materials Institute Lavoisier).

Additionally, the crystallinity of the Pt@MIL-101-Cr materials was examined by means of X-ray powder diffraction (XRPD) measurements. In Figure 1, the XRPD patterns of the pristine MIL-101-Cr and the Pt@MIL-101-Cr materials obtained after the ALD deposition of the Pt nanoparticles using different cycles is presented. The XRPD pattern of each Pt@MIL-101-Cr material presents the pure phase of the non functionalized MIL-101-Cr. This explicitly shows that the framework integrity of the parent MOF was well preserved during the ALD deposition process, despite the use of ozone as reactant. Nitrogen sorption measurements were carried out to determine the Langmuir surface area of the pristine MIL-101-Cr and Pt@MIL-101-Cr materials (see Table 1 and Figure S2 for the nitrogen adsorption isotherms). The MIL-101-Cr has a Langmuir surface area of 3614 m^2^/g, which is significantly higher than the value usually reported in literature because of an extra activation step carried out to remove the free organic linker [34,35]. Only the group of Férey reported a higher Langmuir surface area of approximately 5900 m^2^/g by adding hydrogen fluoride (HF) to the synthesis of the framework [36]. No significant change in the Langmuir surface area is observed for the Pt@MIL-101-Cr materials obtained after 40 cycles and even after 80 ALD cycles. In addition, inspecting the capillary condensation step in Figure S2, it is clear that there is no obvious pore size reduction upon increasing the number of ALD cycles which is in contrast to the work of Snurr, Hupp and Farha [28,37], but, in these cases, metal oxides were prepared by cycling a metal precursor and water in the MOF framework. These adsorption data corroborate the finding that Pt (as a zerovalent metal) is formed as nanoparticles inside the pores, rather than by a layer by layer deposition on the walls, which would result in a gradual pore mouth and cage size reduction. This is further corroborated by tomography and transmission electron microscopy (TEM) data.

#### 2.1.2. TEM Measurements

In order to investigate the Pt loading in the Pt@MIL-101-Cr, (high angle) annular dark field scanning transmission electron microscopy measurements ((HA)ADF-STEM) were carried out on the Pt@MIL-101-Cr-120 cycles sample. As MOFs are known to be extremely sensitive to the electron beam, the electron dose, dwell time and the image magnification were optimized in order to acquire images of the intact MIL-101-Cr framework [38].

**Figure 2:**
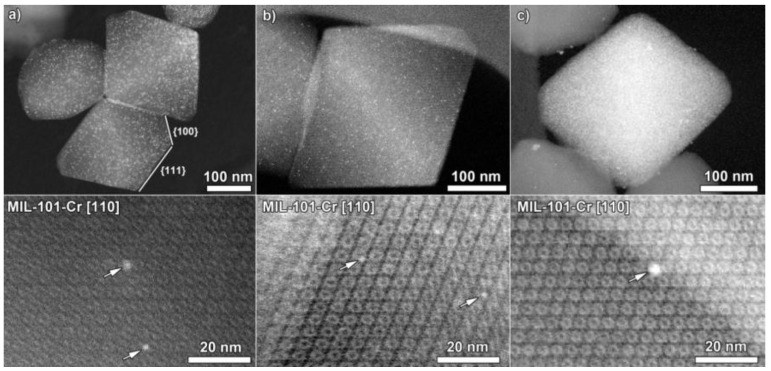
(High angle) annular dark field scanning transmission electron microscopy measurements ((HA)ADF-STEM) (**top row**) and ADF-STEM (**bottom row**) images. (**a**) Fresh Pt@MIL-101-Cr-120 cycles; (**b**) Pt@MIL-101-Cr-120 cycles after run 1; (**c**) Pt@MIL-101-Cr-120 cycles after the high concentration run. The white arrows point to Pt nanoparticles with similar diameters to the MIL-101 framework pores.

The MIL-101-Cr crystals in the Pt@MIL-101-Cr-120 cycles sample demonstrate a typical truncated octahedral morphology, with predominant {111} facets and {100} truncation (Figure 2a). It is clear from the high magnification ADF-STEM images (bottom row in Figure 2) that the MIL-101-Cr crystals maintain their initial crystallinity after the ALD deposition of Pt nanoparticles. The bright contrast features in the images correspond to the heavy Pt nanoparticles which are evenly dispersed in the MIL-101-Cr crystals. The Pt nanoparticle size matches well with the pore diameter of the MIL-101-Cr framework, indicating that they are likely embedded within the pores of the MIL-101-Cr framework. To completely fill the smaller cages with Pt, around 900 Pt atoms are needed, and, in the case of the bigger cages, ~1400 atoms, which is in accordance with the observed Pt NP size of ~2–3 nm. However, the HA(ADF) images are only 2D projections of 3D objects. The direct method to determine the 3D position of the nanoparticles is electron tomography which has been performed on the ALD-loaded Pt@MIL-101 in our previous work. An additional electron tomography series was acquired in this study (see Figure S3 and Movie M.1) on a heavily Pt loaded MIL-101-Cr crystal, which unambiguously demonstrates that the ALD loading of Pt NP into the MIL-101-Cr frameworks leads to the embedding of nanoparticles inside the cages of the MOF host [38]. However, it is also clear from this and our previous study that some of the Pt is remaining at the surface, mostly in the form of larger Pt chunks.

### 2.2. Catalytic Results

A number of reports have already demonstrated the potential of Pt nanoparticles as hydrogenation catalysts. Within this regard, Pt nanoparticles have been immobilized on different supports like carbon nanotubes, silica based materials and MOFs [39,40]. While, in this study, cyclic and linear olefins were utilized as substrates to examine the catalytic performance of Pt@MIL-101-Cr, other studies have used this MOF for the hydrogenation of nitroarenes [41,42], cinnamaldehyde [43] and for the assymetric hydrogenation of α-ketoesters [44]. Additionally, besides these liquid phase based hydrogenation reactions, gas phase olefin hydrogenation reactions have been reported for Pt@MOF catalysts [45,46]. In Table 2, an overview is presented of the investigated substrates employing Pt@MIL-101-Cr-120 cycles as catalyst, compared to some other studies that used Pt@MOF catalysts, while in Figure S4 the conversion patterns using Pt@MIL-101-Cr-120 cycles as catalyst are shown. In Table 3, the TON, TOF and Pt leaching is presented. As can be seen from Table 2, the Pt@MIL-101-Cr-120 cycles exhibits approximately full conversion in the hydrogenation of each examined substrate. For 1-octene full conversion was observed after only 30 min of reaction (entry 5), whereas for styrene (entry 6) 97% conversion was noted after 3 h with the formation of respectively n-Octane and ethyl benzene. However, it is important to note that the blank reactions for the latter substrates already showed a high converison. More specifically, a conversion of 37% of 1-octene was seen after 30 min of reaction whereas, for styrene, 50% was already converted after 3 h of reaction in the absence of the catalyst. For the other examined substrates, cyclohexene and cyclooctene, the conversions obtained for the blank reactions were significantly lower with only 11% cyclohexene conversion after 2 h of reaction time and no conversion of cyclooctene under these reaction conditions. In the presence of the catalyst, 94% of cyclooctene was converted after 6 h (entry 7), whereas, for cyclohexene, 98% of conversion was noted after just two hours of reaction (entry 8). The latter substrate can also be converted under solvent free conditions (entry 9). Full conversion was noted after 20 h of reaction.

Additionally, the Pt@MIL-101-Cr-120 cycles’ catalyst was compared with other Pt@MOF based heterogeneous catalysts for the hydrogenation of linear and cyclic olefins. Although it is difficult to give an objective comparison, as different catalytic conditions were used in these tests, it can be seen from Table 2 that each Pt based catalyst, including the Pt@MIL-101-Cr-120 cycles, exhibits a good catalytic performance in the hydrogenation of alkenes except for the Pt@ZIF-8 (ZIF = zeolitic imidazolate framework) in the hydrogenation of cyclooctene. For the substrate 1-hexene, using Pt@ZIF-8, 95% of conversion in 24 h was obtained with no side product formation, whereas almost no activity was seen in the hydrogenation of cyclooctene (2.7%) (entry 2 and 3). The authors adressed this difference in activity to the difference in size of the examined substates, as cyclooctene has a molecular width of 5.7 Å which exceeds the size of the apertures of ZIF-8 (3.4 Å) [48]. The pore aperture in MIL-101-Cr is significantly larger (12 and 15 Å), as MIL-101-Cr contains two types of cages having a diameter of respectively 29 Å and 34 Å. The enhanced catalytic performance of the Pt@MIL-101-Cr-120 cycles catalyst in comparison to the reported Pt@ZIF-8 for the hydrogenation of cyclooctene can be assigned to this difference in pore aperture. For cyclohexene, styrene and cyclooctene the turnover frequency (TOF) is respectively 4.4 min^−1^, 3.7 min^−1^ and 1.93 min^−1^, as can be expected as the kinetics slow down as the molecules become larger. Furthermore, the room temperature based conversion of styrene in this work, in a very recent study of Li *et al.* [49], the latter substrate was fully converted at the same reaction time (3 h) under a H_2_ pressure of 1 bar through use of a bimetallic Pt-Ni frame@MOF-74, but no product distribution was presented (entry 4).

### 2.3. Reusability and Stability Tests

**Figure 3:**
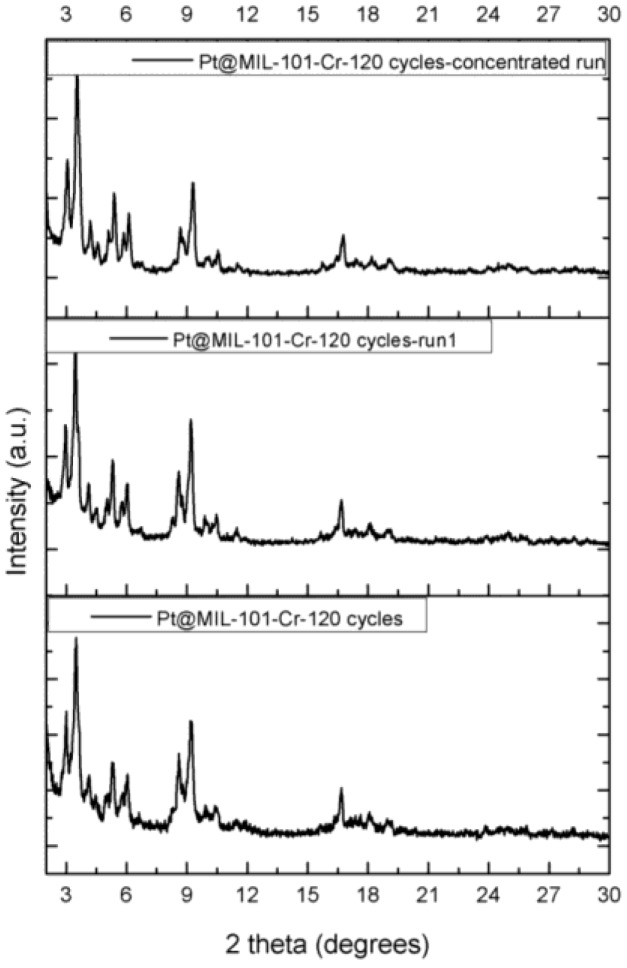
XRPD pattern of the pristine catalyst and after the first run and the concentrated run.

To examine the reusability of the Pt@MIL-101-Cr-120 cycles catalyst, a high concentration run was carried out in which 10 times more substrate was added in comparison to the previous catalytic experiments, without changing the catalyst loading. This procedure is employed when little catalyst is available, as repeated filtration steps result in cumulative catalyst losses. In addition, 250 Mmol of cyclooctene was added into the Parr reactor and the reaction was monitored until full conversion was obtained. As can be seen from Figure S5, nearly full conversion was reached after approximately 168 h of reaction. This observation demonstrates that the Pt@MIL-101-Cr-120 cycles catalyst does not lose its activity nor becomes deactivated during many turnovers. Additionally, during this high concentration run, only a negligible amount of Pt NPs was leached out: 0.81% of Pt was leached from the Pt@MIL-101-Cr-120 cycles. The turnover number TON (determined at the end of the reaction) and TOF (determined after 2 h of catalysis) number for this concentrated run is respectively 4859 and 108 h^−1^. Comparison of the XRPD patterns of the Pt@MIL-101-Cr-120 cycles before and after catalysis clearly shows that no changes are observed in the XRPD pattern of the Pt@MIL-101-Cr-120 cycles after catalysis when compared to the pristine MOF, even after the high concentration run (see Figure 3). The latter observation shows that the framework integrity of the MOF was preserved.

Additionally, in Figure S6, the nitrogen adsorption isotherms are presented for the Pt@MIL-101 material before and after catalysis. From this figure, one can see that the Langmuir surface area slightly decreased after catalysis, which is probably due to a partial clogging of the pores during the catalytic testing. The fresh catalyst has a Langmuir surface area of 3210 m^2^/g, the Pt@MIL-101-Cr-120 cycles-run 1 and Pt@MIL-101-Cr-120 cycles-concentrated run have a Langmuir surface area of 2650 and 2700 m^2^/g respectively. Moreover, annular dark field scanning transmission electron microscopy measurements were carried out on the Pt@MIL-101-Cr-120 cycles after run 1 (Figure 2b) and after the high concentration run (Figure 2c). It can be seen from these images that the morphology of the MIL-101 particles remains similar to these of the fresh Pt@MIL-101-Cr-120 cycles sample and that the MIL-101 framework retains its crystalline nature. Additionally, no significant agglomeration of the Pt nanoparticles is observed, even after the high concentration run.

## 3. Experimental Section

### 3.1. Materials and Methods

All chemicals were purchased from Sigma Aldrich (Diegem, Belgium) or TCI Europe (Zwijndrecht, Belgium) and used without further purification. Nitrogen adsorption experiments were carried out at −196 °C using a Belsorp-mini II gas analyzer (Rubotherm, Bochum, Germany). Prior to analysis, the samples were dried under vacuum at 90 °C to remove adsorbed water. XRPD patterns were collected on a ARL X’TRA X-ray diffractometer (Thermo Fisher Scientific, Erembodegem, Belgium) with Cu Ka radiation of 0.15418 nm wavelength and a solid state detector. XRF measurements were performed on a NEX CG from Rigaku (Addspex, Abcoude, the Netherlands) using a Mo-X-ray source (Addspex, Abcoude, the Netherlands) Elemental analyses was conducted using a Vista-MPX CCD Simultaneous Inductively Coupled Plasma-Optical Emission Spectrometer (ICP-OES) (Waltham, MA, USA). HAADF-STEM and ADF-STEM imaging was carried out on a FEI Tecnai Osiris microscope (Hillsboro, Oregon, USA), operated at 200 kV. The convergence semi-angle used was 10 mrad, the inner ADF detection angle was 14 mrad, the inner HAADF-STEM detection angle was 50 mrad.

### 3.2. Catalytic Setup

In each catalytic test, the Parr reactor was loaded with 70.0 mL ethanol and 2.84 mL of dodecane used respectively as solvent and internal standard. The examined substrates in this study are 1-octene, styrene, cyclooctene and cyclohexene (25 mmol). The Pt@MIL-101-Cr-120 cycles was used as the catalyst. For each examined substrate, the same loading of active sites was employed. More specifically in every catalytic test, 0.05 mmol Pt sites were used which give rise to a molar ratio of substrate: catalyst of 500:1. The TON number was calculated by dividing the mmol obtained product by the number of active sites while the TOF number was determined by dividing the TON number by the reaction time (expressed in minutes). All the catalytic tests were performed at room temperature and at a pressure of 6 bar H_2_. During each test, aliquots were gradually taken out of the mixture and subsequently analyzed by means of gas chromatography (GC) using a split injection (ratio 1:17) on a Hewlett Packard 5890 Series II GC with TCD detection (Santa Clara, CA, USA). The capillary column used was a Restek XTI-5 column (Bellefonte, PA, USA) with a length of 30 m, an internal diameter of 0.25 mm and a film thickness of 0.25 μm. H_2_ was used as carrier gas under constant flow conditions (1.4 mL/min).The fresh catalyst was activated under vacuum at 90 °C overnight prior to catalysis. After each catalytic run, the catalyst was recovered by filtration, washed with acetone, and dried at 90 °C overnight under vacuum.

### 3.3. Synthesis of MIL-101-Cr and Pt@MIL-101-Cr

The MIL-101-Cr was synthesized according to a slightly modified procedure of Edler *et al.* [34]. Typically, 0.6645 g of terephthalic acid was mixed with 1.6084 g of Cr(NO_3_)_3_·9H_2_O and 20 mL of destilled water. The mixture was transferred into a Teflon-lined autoclave, sealed and heated in 2 h to 210 °C at which it was hold for 8 h. After cooling down to room temperature, the MIL-101*as* was filtered, washed with acetone and stirred in dimethylformamide for 24 h at 60 °C to remove unreacted terephthalic acid. Thereafter, the MIL-101 was stirred in 1M HCl for 12 h at room temperature, filtered and dried under vacuum at 90 degrees overnight. The deposition of the Pt nanoparticles was achieved by means of ALD. Pt ALD on the powder sample was performed at 200 °C using (methylcyclopentadienyl)-trimethylplatinum [MeCpPtMe_3_] as Pt source and O_3_ as reactant [50]. All the depositions were conducted in a home built experimental cold-wall ALD chamber connected through a gate valve to a turbo pump backed up by a rotary pump. A second gate valve was installed for pre-evacuation of the chamber via a bypass line to the rotary pump. The powder sample was loaded in a molybdenum sample cup which was then transferred into the ALD reactor through the load-lock and was placed on a heater block. After loading into the reactor, the powder sample was allowed to outgas and thermally equilibrate for at least 1 h under vacuum. The solid MeCpPtMe_3_ precursor (99% Strem Chemicals), kept in a stainless steel container, was heated above its melting point (30 °C), and the delivery line to the chamber was heated to 60 *°*C. Argon was used as a carrier gas for the Pt precursor. O_3_ was produced from a pure O_2_ flow with an OzoneLab™ OL100 ozone generator (Ozone Services, Burton, BC, Canada), resulting in an O_3_ concentration of 175 μg/mL. A static exposure mode was applied during both ALD half-cycles [1,4]. The pulse time of the MeCpPtMe_3_ precursor was 10 s, after which the valves to the pumping system were kept closed for another 20 s, resulting in a total exposure time of 30 s. The same pulse time and exposure time was used for the O_3_ also. The effect of the exposure times on the Pt loading was not studied in detail. Nevertheless, these exposure times were found to be large enough to ensure penetration deep into the MOF crystals. During the precursor and reactant exposures, the pressure in the chamber increased to ca. 5 × 10^−1^ mbar and 1 mbar, respectively. In between the two exposures, the valve to the rotary pump was first opened for 10 s and then the valve to the turbo pump was opened for another 50 s to reach the base pressure.

## 4. Conclusions

Pt NPs were synthesized *in situ* within MIL-101-Cr by means of ALD, enabling the varying of Pt loading by changing the number of ALD cycles. Highly dispersed Pt NPs were obtained with sizes determined by the pore sizes of the MOF host. The Pt@MIL-101 materials maintained their porosity and crystallinity during the synthesis of the Pt NPs and during the catalytic hydrogenation of cyclic and linear olefins. Full conversion for every substrate was obtained using Pt@MIL-101 as catalyst under mild reaction conditions. Moreover, even under solvent free conditions, full conversion was shown with negligible leaching of Pt. Stability tests have demonstrated that the Pt@MIL-101 catalyst is stable for a long reaction time without loss in crystallinity or agglomeration of the Pt NPs, and with a high TOF and TON number.

## Figures and Tables

**Table 1:** Langmuir surface area (S_lang_) and Pt loading of the Pt@MIL-101-Cr materials (MIL = Materials Institute Lavoisier).

Sample	Pt Loading (mmol·g^−1^)	S_lang_ (m^2^·g^−1^)	Pore volume (cm^3^ g^−1^) *
MIL-101-Cr	/	3614	1.52
Pt@MIL-101-Cr-40 cycles	0.21	3418	1.47
Pt@MIL-101-Cr-80 cycles	0.3	3304	1.48
Pt@MIL-101-Cr-120 cycles	0.35	3210	1.42

* After normalization for the amount of the Pt, determined at a relative pressure P/P_0_ = 0.98.

**Table 2:** Comparison of the catalytic activity of Pt@MIL-101-120 cycles with other Pt based Metal Organic Frameworks (MOF) catalysts in the hydrogenation of cyclic and linear olefins.

Entry	Catalyst	Substrate	Reaction Conditions	Reaction Time	Conversion	Main Product	Reference
1	Pt@MIL-101	1-octene	35 °C, solvent free at 1.5 bar of H_2_	6 h	>99%	n-Octane	[47]
2	Pt@ZIF-8	1-hexene	RT, ethanol at 1 bar of H_2_	24 h	>95%	n-Hexane	[48]
3	Pt@ZIF-8	cyclooctene	RT, ethanol at 1 bar of H_2_	24 h	2.7%	Cyclooctane	[48]
4	Pt-Ni frame@ Ni-MOF-74	Styrene	30 °C, THF at 1 bar of H_2_	3 h	>99%	/	[49]
5	Pt@MIL-101	1-octene	RT, ethanol at 6 bar of H_2_	30 min	>99%	n-Octane	this work
6	Pt@MIL-101	Styrene	RT, ethanol at 6 bar of H_2_	3h	>97%	Ethyl benzene	this work
7	Pt@MIL-101	cyclooctene	RT, ethanol at 6 bar of H_2_	6h	>94%	Cyclooctane	this work
8	Pt@MIL-101	cyclohexene	RT, ethanol at 6 bar of H_2_	2h	>98%	Cyclohexane	this work
9	Pt@MIL-101	cyclohexene	60 °C, solvent free at 6 bar of H_2_	20h	>99%	Cyclohexane	this work

**Table 3:** The turnover number (TON), turnover frequency (TOF) and leaching percentage for each examined substrate using Pt@MIL-101-Cr-120 cycles as catalyst.

Substrate	TON	TOF (min^−1^)	Reaction Time	Leaching of Pt (%)
1-Octene	497	16.6	30 min	<0.05 *
Styrene	482.7	3.7	3h	0.89
Cyclohexene	490	4.4	2h	0.32
Cyclooctene	468	1.93	6h	<0.05 *

* Below detection limit. The TON number was determined at the end of the reaction while the TOF number was determined after 30 min of catalysis.

## Supplementary Materials

The following are available online at http://www.mdpi.com/2079-4991/6/3/45/s1.
