# Supplementary material for: Atomic Layer Deposition of Pt Nanoparticles within the Cages of MIL-101: A Mild and Recyclable Hydrogenation Catalyst
Source: Nanomaterials (Basel). 2016 Mar 9;6(3):45. doi: 10.3390/nano6030045 (PMC5302512; doi:10.3390/nano6030045)
Supplement: Supplementary file 1 [file nanomaterials-06-00045-s001.zip › nanomaterials-118769-supplementary-final.pdf]

Supplementary Materials: Atomic Layer Deposition of Pt Nanoparticles within the Cages of MIL-101: A Mild and Recyclable Hydrogenation Catalyst

Karen Leus [1,*], Jolien Dendooven [2], Norini Tahir [1], Ranjith K Ramachandran [2], Maria Meledina [3], Stuart Turner [3], Gustaaf Van Tendeloo [3], Jan L. Goeman [4], Johan Van der Eycken [4], Christophe Detavernier [2] and Pascal Van Der Voort [1,*]

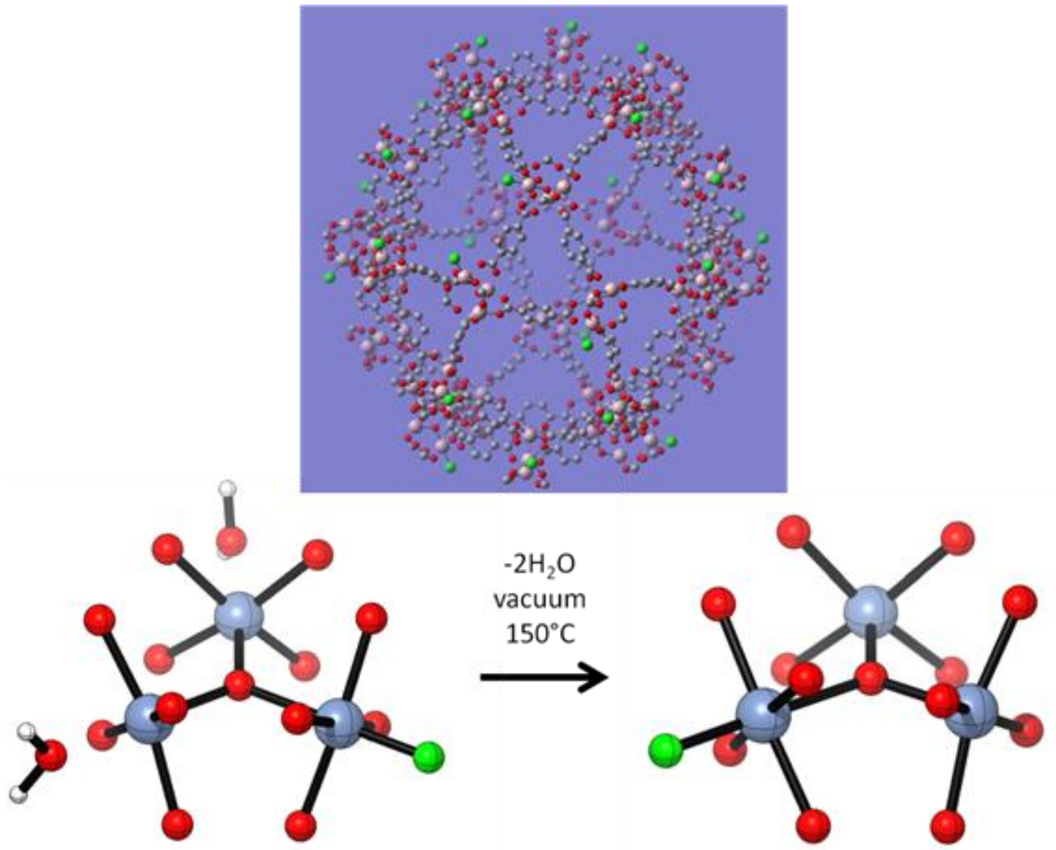

Figure S1. Schematic structure of a MIL-101 cage (**top**) and formation of coordinatively unsaturated sites after the removal of the terminal water molecules (**bottom**). (MIL = Materials Institute Lavoisier).

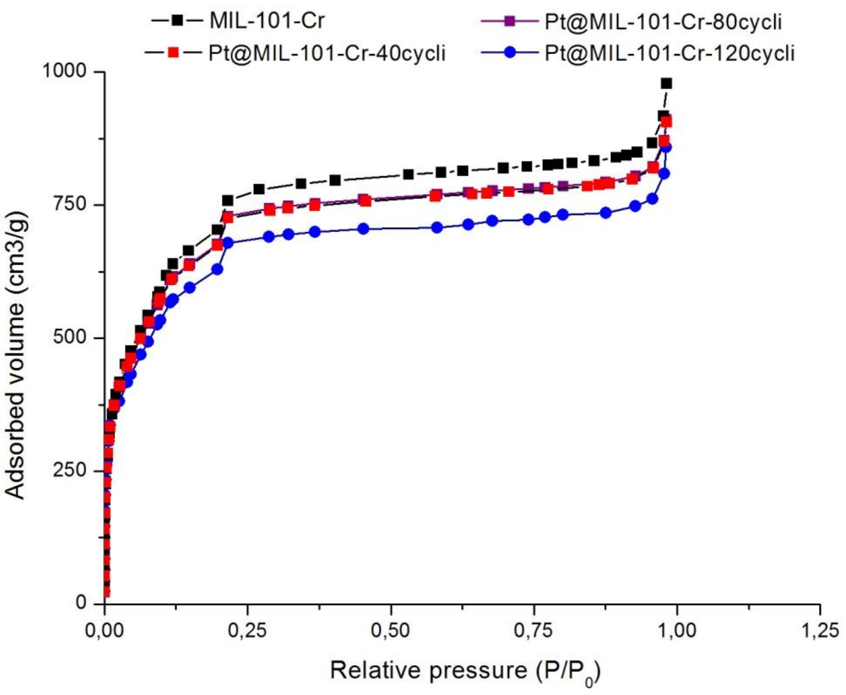

Figure S2. Nitrogen adsorption isotherms for MIL-101-Cr and the Pt@MIL-101-Cr obtained after respectively 40, 80 and 120 atomic layer deposition (ALD) cycles.

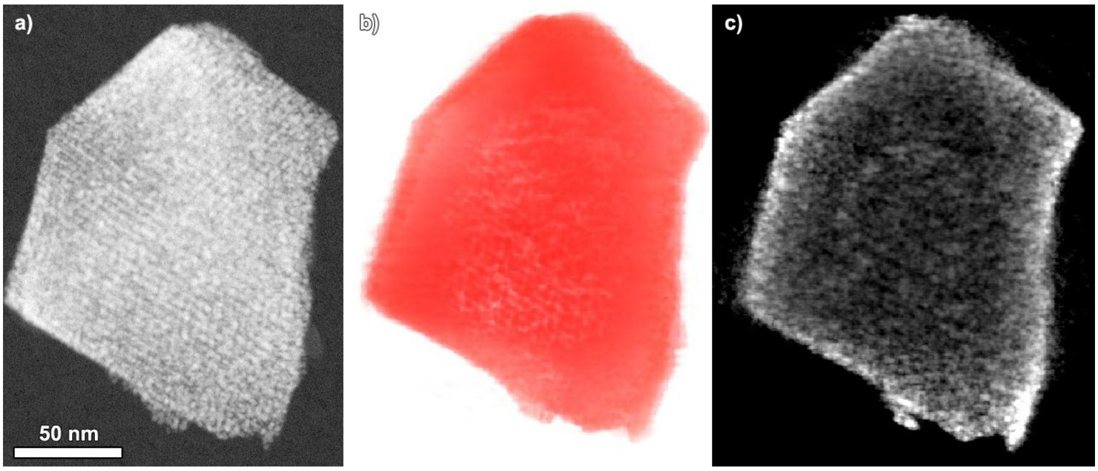

Figure S3. (**a**) (high angle) annular dark field scanning transmission electron microscopy measurements ((HA)ADF-STEM) image of a MIL-101 particle heavily loaded with Pt nanoparticles; (**b**) tomographic volume reconstruction of the same Pt@MIL-101 crystal and (**c**) orthoslice through the reconstruction.

See also uploaded movie.

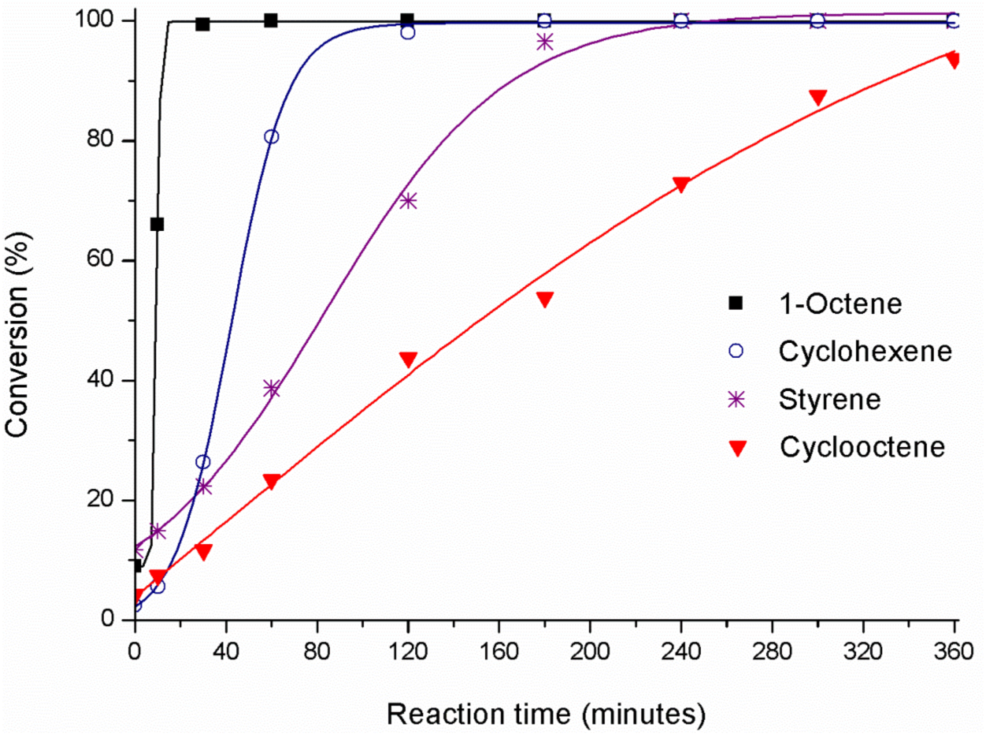

Figure S4. Conversion patterns using Pt@MIL-101-Cr-120 cycles as a catalyst and 1-octene (**black**), cyclohexene (**blue**), styrene (**purple**) or cyclooctene (**red**) as the substrate. Reaction conditions: 25 mmol of substrate, 0.05 mmol of catalyst, room temperature, 6 bar of H_2.

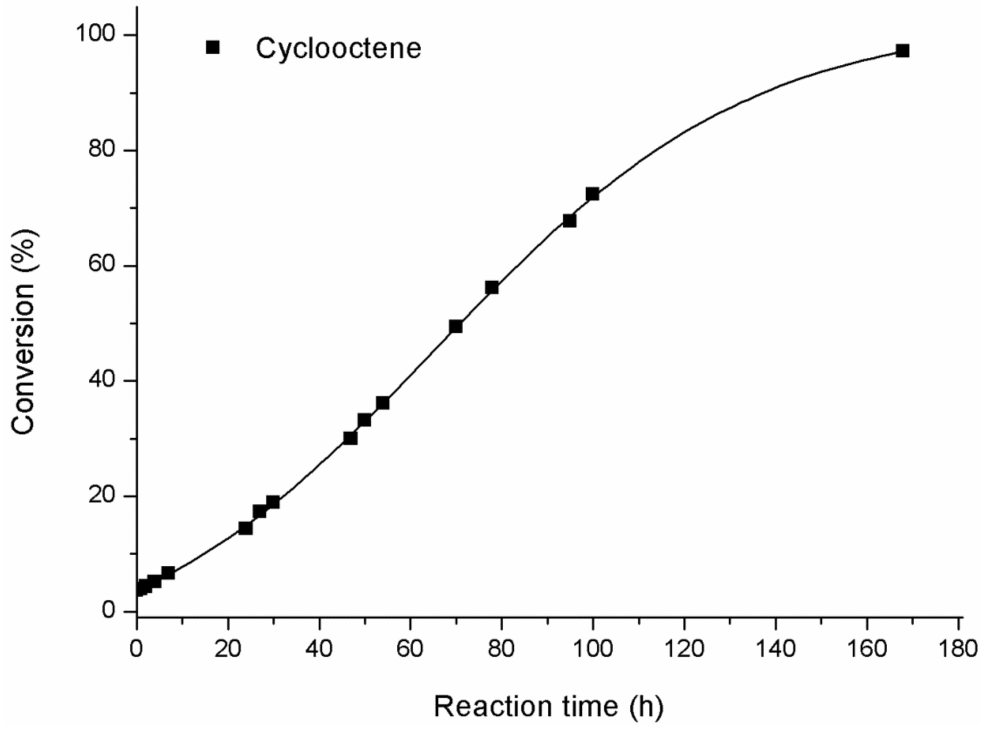

Figure S5. Concentrated run using Pt@MIL-101-Cr-120 cycles as a catalyst and cyclooctene as the substrate. Reaction conditions: 250 mmol of cyclooctene, 0.05 mmol of catalyst, room temperature (RT), 6 bar of H_2.

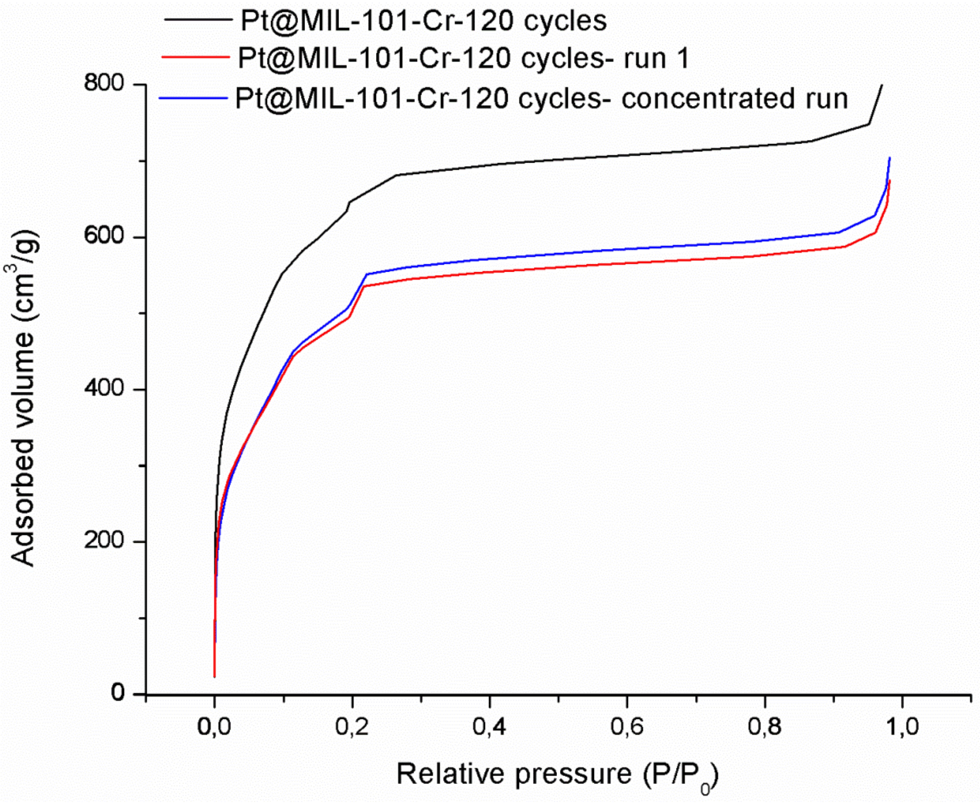

Figure S6. Nitrogen adsorption isotherms of Pt@MIL-101-Cr-120 cycles before (**black**) and after the first catalytic run (**red**) and the concentrated catalytic test (**blue**).

© 2016 by the authors; licensee MDPI, Basel, Switzerland. This article is an open access article distributed under the terms and conditions of the Creative Commons by Attribution (CC-BY) license (http://creativecommons.org/licenses/by/4.0/).
